# Supplementary figures and images for: Reproductive benefits and reduced investment in parental care behavior associated with reproductive groups of males in Abudefduf troschelii
Source: PeerJ. 2023 Aug 7;11:e15804. doi: 10.7717/peerj.15804 (PMC10414026; doi:10.7717/peerj.15804)

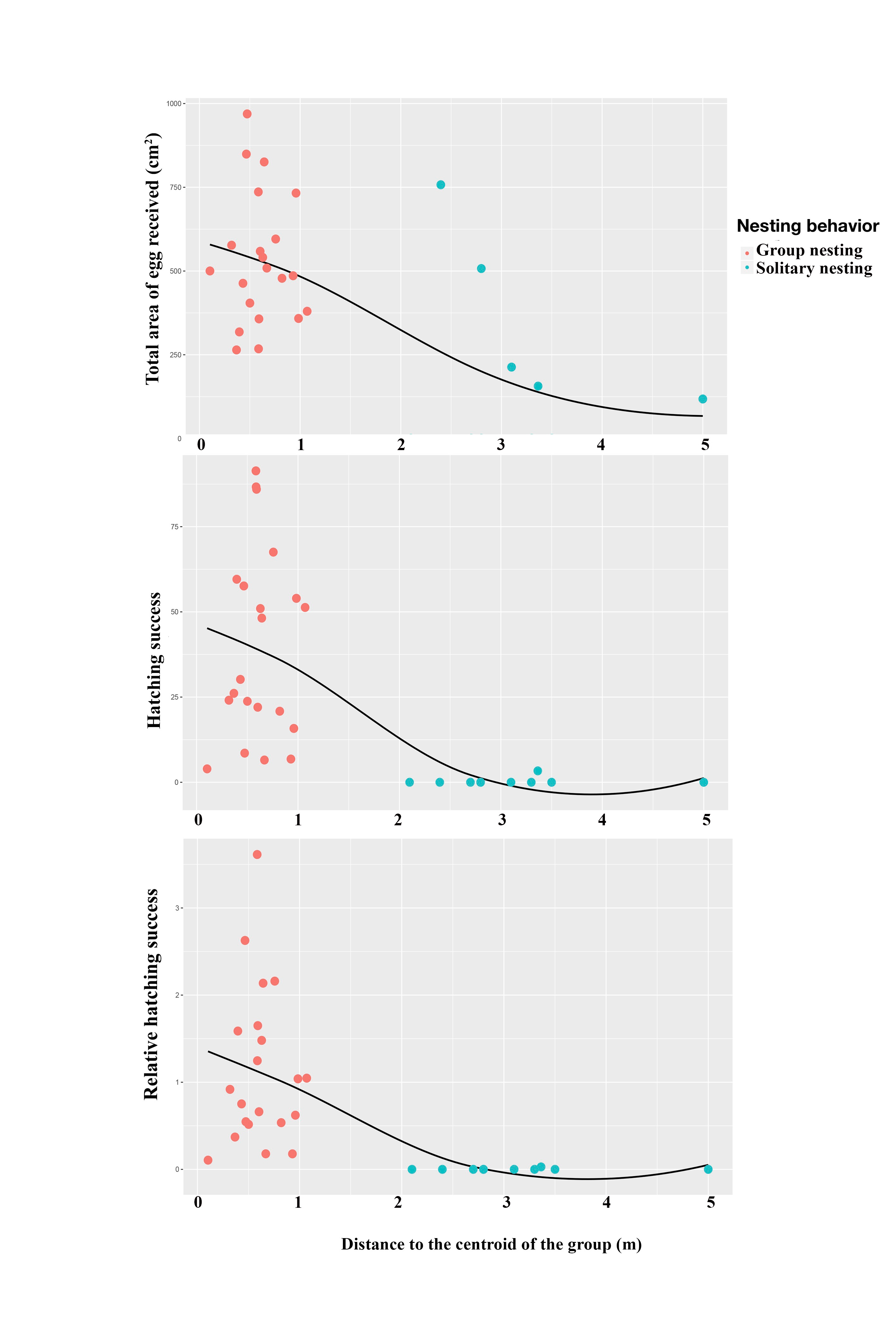

Supplement: Supplemental Information 1 [file peerj-11-15804-s001.png]

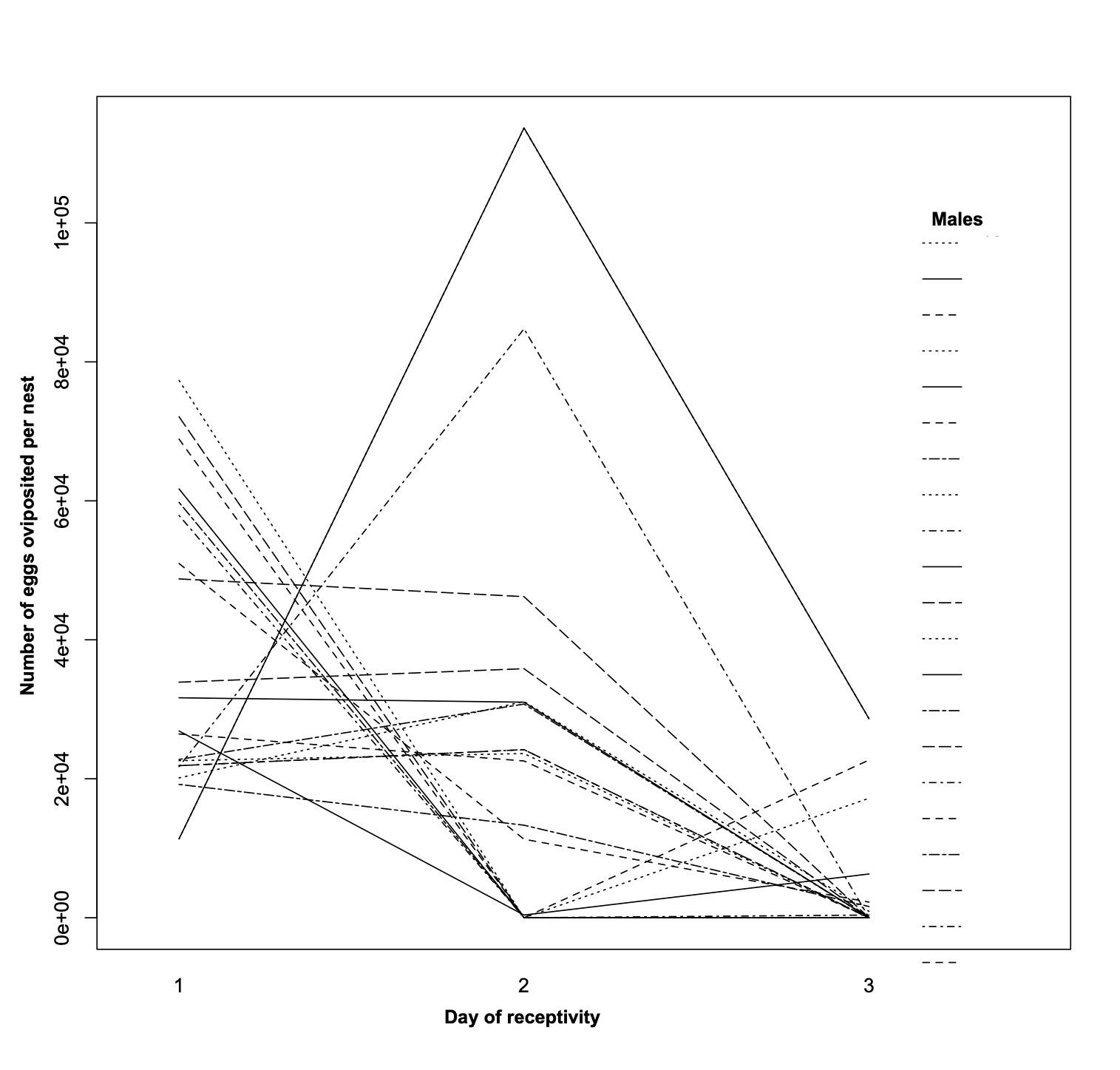

Supplement: Supplemental Information 2 [file peerj-11-15804-s002.png]

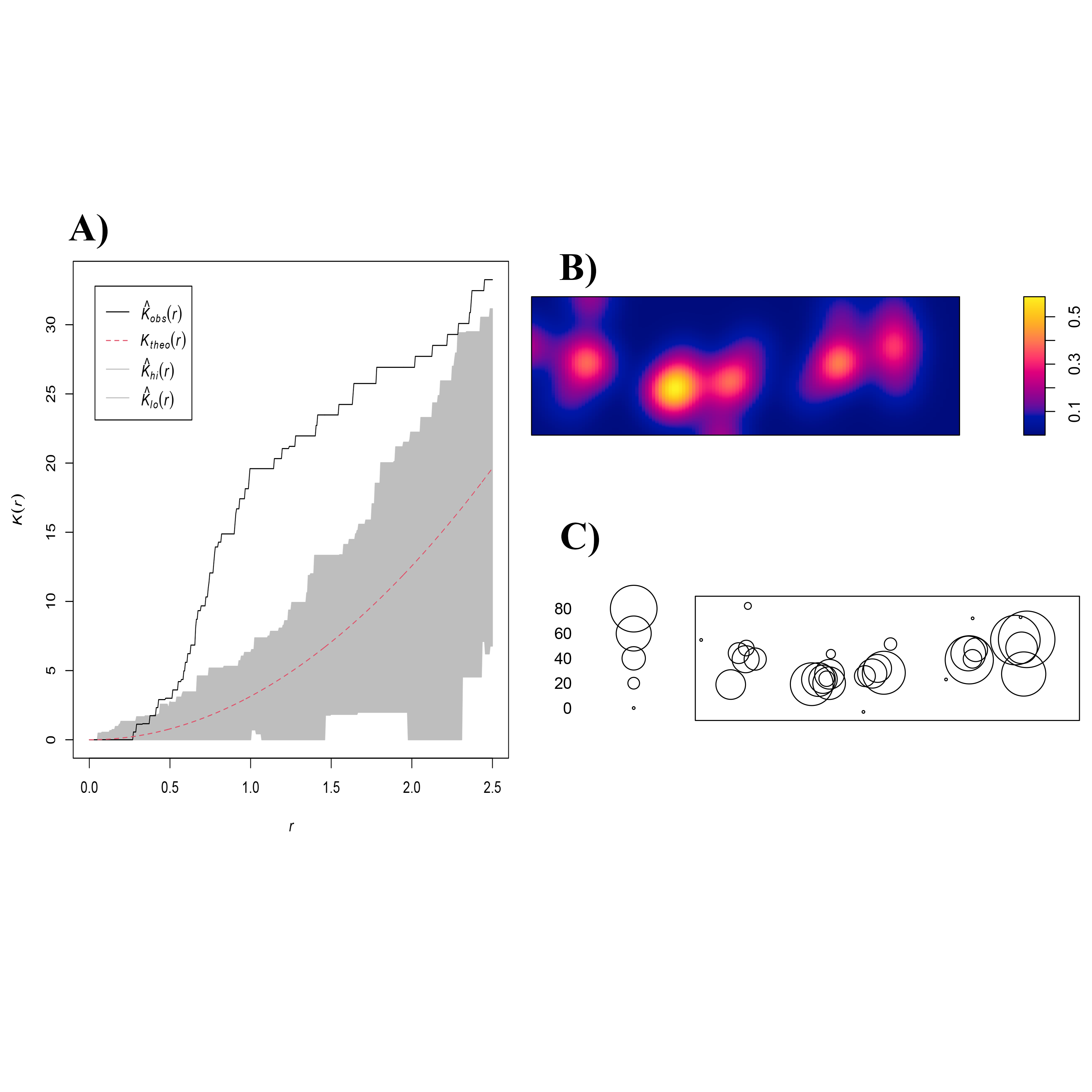

Supplement: Supplemental Information 3 [file peerj-11-15804-s003.png]
